# University Students’ Notion of Autism Spectrum Conditions: A Cross-Cultural Study

**DOI:** 10.1007/s10803-019-04343-z

**Published:** 2020-01-03

**Authors:** Marieke de Vries, Sabrina Cader, Lucy Colleer, Eleonore Batteux, Meryem Betul Yasdiman, Yih Jiun Tan, Elizabeth Sheppard

**Affiliations:** 1 School of Psychology, University of Nottingham Malaysia Campus, Jalan Broga, 43500 Semenyih, Selangor Malaysia; 2 School of Psychology, University of Nottingham, Nottinghamshire, UK

**Keywords:** Culture, Attitudes acceptance, Interaction, Beliefs

## Abstract

Cultural background might influence knowledge and attitudes regarding autism, influencing willingness to interact. We studied whether beliefs, knowledge, contact, and attitude differed between the UK and Malaysia. With mediation analyses, we studied how these factors influenced willingness to interact. Autism was more often linked to food in the UK, and to upbringing in Malaysia. Knowledge, contact, and acceptance were greater in the UK. When excluding psychology students, Malaysian students were less willing to interact with autistic people. Knowledge and contact appeared to improve acceptance, but acceptance did not mediate the relation between country, beliefs, knowledge, and experience; and willingness to interact. Knowledge and contact regarding autism might improve acceptance in different cultures, but how acceptance could improve interaction is unclear.

Autism spectrum disorder (ASD) is a neurodevelopmental disorder characterised by impairments in social interaction and communication, and rigid and stereotyped behaviour (American Psychiatric Association 2013). There is an increase in prevalence of ASD diagnoses over the last decades (Russell et al. 2014), which can be partly explained by increasing awareness (Fombonne 2018). With increased awareness, and knowledge about Autism Spectrum Conditions (ASC; conditions associated with ASD), stigma seems to decrease. However, knowledge and beliefs about ASC differ between countries and cultures, and there is still a lack of awareness about, and a stigma on, ASC in many cultures (Obeid et al. 2015).

Stigma consists of labeling, stereotyping, separation, status loss, and discrimination (Link and Phelan 2001), and depends on power, i.e., people who stigmatise have, in some way, power over the stigmatised group. Although a stigmatized group can be described as passive, or helpless victims, often, there is resistance against stigma (Link and Phelan 2001), but as power is involved, it is difficult to battle stigma. Outcomes of stigma can be drastic and vary widely (Link and Phelan 2001), but include stress, poorer work opportunities, and living conditions. Both individuals with ASC, and their family members are often stigmatized (blaming, assuming contamination, and pity) (Milacic-Vidojevic et al. 2014). Moreover, stigma might internalise, resulting in self-stigma, causing severe psychological distress (Ali et al. 2012). In parents of children with ASC, self-stigma is related to feeling a lack of control over their child’s condition, behaviour and stigma, and self-blame (Mak and Kwok 2010). In short, stigma has serious negative consequences for ASC families.

People with the least knowledge about ASC tend to stigmatise the most (Milačić-Vidojević et al. 2014), hence, knowledge might decrease stigma. However, the attribution model of stigma states that not only knowledge about a condition, but also attribution of controllability influences the affective response and stigma towards someone with a certain condition (Ling et al. 2010), such as ASC. Perceived low controllability (the person with the condition is not accountable for or has no control over the condition or his/her behaviour) might induce positive emotions (sympathy and helping behaviour), while perceived high controllability (the person with the condition is considered accountable) might induce negative emotions (anger and punishing behaviour) (Ling et al. 2010). Although knowledge about and experience with ASC seems to reduce negative behaviour (punishing intention in teachers), it does not necessarily increase helping behaviour. Helping behaviour increases with perceived less controllability and more sympathy (Ling et al. 2010). Hence, knowledge alone might not be enough to reduce stigma. Beliefs about ASC, such as perceived controllability, and affective response influence attitudes towards ASC as well.

Culture influences knowledge and beliefs about ASC (Obeid et al. 2015). For example, collectivism/individualism tendencies seem to influence stigma and attitudes towards mental illness. Stigma seems higher in collectivistic cultures (Papadopoulos et al. 2013), as compared to individualistic cultures. This might result from less acceptance of diversity in collectivistic cultures (Papadopoulos et al. 2013). Access to services differs between countries and might additionally influence knowledge and stigma. In many non-western countries, such as Malaysia, families from, and individuals with an ASC have limited access to diagnostic centers, interventions and support (e.g., Toran et al. 2013). Although there is an increase in ASC centers in the urban areas, rural areas remain poorly equipped. Moreover, the ASC centers appear mostly accessible to the more fortunate and have long waitlists. The less fortunate and people in rural areas might rely more on the alternative circuit (Lim 2015). One might expect that knowledge and beliefs about ASC differs between people in countries with limited (access to) ASC resources as compared to countries with good resources. This might influence stigma, attitudes and willingness to interact.

Although stigma and attitudes towards ASC have been studied (e.g., Ali et al. 2012; Gardiner and Iarocci 2014; Sasson et al. 2017), only two studies directly compared two culturally different countries, i.e., the United States to Lebanon (Obeid et al. 2015) and to Japan (Someki et al. 2018). As misconceptions and stigma towards ASC differ between countries (Obeid et al. 2015; Someki et al. 2018), it is important to study how these beliefs, knowledge, and experience with individuals with ASC influence peoples’ attitude towards and willingness to interact with someone with ASC in culturally different countries. With the current study, we want to extend these previous findings by studying attitudes towards ASC in the UK and Malaysia, and explicitly study the willingness to interact with someone with ASC.

Willingness to interact with someone with ASC is particularly important, as social interaction poses a challenge for individuals with ASC. Besides the social challenges they encounter themselves, social partners might find it difficult to identify the mental state of someone with ASC (Edey et al. 2016; Sheppard et al. 2016). Moreover, when unaware of the diagnosis, based on very limited (audio and/or visual) information, students seem less willing to interact with peers with ASC than with peers without ASC (Sasson et al. 2017). Students perceive peers with ASC as more awkward and less approachable, hence might respond less favorably to individuals with ASC than without ASC (Sasson et al. 2017). Hence, the social difficulties that people with ASC encounter seem only partly resulting from their own behaviour, but also from their social partners.

The transition from childhood into adulthood is a challenging phase for individuals with ASC (Eaves and Ho 2008). The attitude of typically developing peers towards students with ASC can contribute essentially to participation of individuals with ASC at their university (Gardiner and Iarocci 2014). Families of young adults with ASC report that there is a great need for social outlets, friends, work and education, while attitudes and ignorance are considered unhelpful (Eaves and Ho 2008). Knowledge about and experience with ASC might help to increase positive attitudes towards individuals with ASC and improve the social life of individuals with ASC, although factors like beliefs and affect (Ling et al. 2010) seem to play a role as well. The question is whether this also applies to students, and whether this is similar across cultures.

In short, individuals with ASC have social difficulties, and typically developing peers play a significant role in social interaction (Sasson et al. 2017). Knowledge, beliefs and experience influence the willingness to interact with someone with ASC (Gardiner and Iarocci 2014), but the relation between culture, knowledge, beliefs, and acceptance is still unclear. Therefore, we aimed to pin down; (1) whether beliefs, knowledge and experience with ASC differ between students in the UK and Malaysia; (2) how beliefs, knowledge and experience with ASC influence acceptance and willingness to interact with someone with ASC in the UK and Malaysia.

The UK and Malaysia are excellent countries to examine cultural effects in a rather comparable student population. English is widely spoken in Malaysia, hence questionnaires can be administered in the English language. Moreover, private university students are well comparable to UK students with respect to socio-economic status. Nevertheless, there are several cultural differences. Besides the obvious location of the countries (Southeast Asian vs Western European), the economy is rather different. The UK is considered a developed country, and Malaysian economy is rapidly developing (Toran et al. 2011). Furthermore, the UK is individualistic; Malaysia is collectivistic (Tafarodi and Smith 2001). This is reflected in the nature and availability of (mental) health care. In the UK the first step towards (mental) health care is usually the general practitioner and most people have access to (mental) healthcare. Children are monitored according to the Healthy Child Program (HCP), including regular checkups (Wolfe et al. 2016), and health visitors providing parents with information regarding (mental) health. In Malaysia, family is considered extremely important and ethnic and religious background is valued highly. For information and support with respect to (mental) health, many are dependent on informal sources, such as (extended) family (Toran et al. 2013). Not everyone has access to (mental) health care facilities and there is a shortage of personnel, such as psychiatrists (Haque 2005). In short, the UK and Malaysia have a different culture and a different health care system, which might influence what people know about, and how they feel about ASC.

We expect that beliefs, knowledge and experience with ASC differ between the UK and Malaysia; we expect that students in the UK have more knowledge and experience with ASC, as ASC awareness appears higher in the UK compared to Malaysia. We expect that these differences in knowledge, the beliefs about ASC, and experience with ASC will influence acceptance and in turn the willingness to interact with someone with ASC.

To report the influence from the current living environment (the UK or Malaysia) of the students, the main analyses include all participating students, disregarding nationality. Given the large number of international students, additionally we will repeat analyses including only British and Malaysian students, to explore the influence of specific cultural background. Finally, we will explore the influence of psychology students in the sample.

## Methods

### Participants

Students from the University of Nottingham UK (*N *= 166) and Malaysia (*N* = 195) participated in the study. Table 1 shows demographic details of the participants including gender, age, and field of study at each location. As expected, the majority of participants in the UK were British, and the majority in Malaysia was Malaysian. However, students from other countries were not excluded at either campus and both samples included a range of additional nationalities. In total, the UK sample included 108 British, 5 Chinese, 4 Saudi, 3 Turkish, 3 Spanish, 3 Malaysian, 2 Russian, 2 Mexican, 2 Nigerian, 2 German, 2 Cypriot, 2 American, 2 Polish, 2 Danish, 1 Latvian, 1 Azerbaijani, 1 Czech, 1 Icelandic, 1 Ghanaian, 1 Taiwanese, 1 Chilean, 1 Faroese, 1 Thai, 1 Mauritian, 1 Singaporean, 1 Slovak, 1 Kuwaiti, 1 Indian, 1 Tanzanian, 1 Motswana, 1 Kenyan, 1 French, 1 Indonesian, 1 Portuguese, and 4 participants who did not state their nationalities. The Malaysian sample included 130 Malaysians, 18 Sri Lankans, 9 Mauritians, 8 Pakistanis, 4 Indians, 4 Singaporeans, 3 Kenyans, 3 British, 2 Americans, 2 Egyptians, 1 Indonesian, 1 South Korean, 1 Tanzanian, 1 Seychellois, 1 Bruneian, 1 Italian, 1 Zambian, 1 Bangladeshi, 1 Polish, 1 Nigerian, 1 Vietnamese, and 1 Sudanese.

**Table 1:** Socio demographics of the UK and Malaysia student populations

	UK (*N *= 166)	Malaysia (*N *= 195)
Gender		
Male	69 (41.6%)	78 (40%)
Female	94 (56.6%)	117 (60%)
Other	1 (0.60%)	0
Did not state	2 (1.20%)	0
Age (*M, S.D.)*	22.00 (4.37)	20.61 (1.78)
Subject		
Arts & Humanities	44 (26.5%)	12 (6.2%)
Social Sciences and Law	24 (14.4%)	57 (29.2%)
Science and Medicine	70 (42.2%)	76 (39.0%)
Engineering	17 (10.2%)	43 (22.1%)
Did not state	11 (6.6%)	7 (3.6%)

Given the variability in nationalities within the samples, additional more focused analyses were carried out examining the British and Malaysian participants’ responses only. Moreover, we examined the effect of field of study on the responses as the proportions studying in each area differed somewhat between the two campuses.

### Procedure

Participants were recruited from various public spaces around the campuses (e.g., libraries, cafeterias, common areas), to include a wide range of academic disciplines. The study was not advertised to avoid participation bias by students who had a particular interest in or knowledge of ASC. Potential participants were approached by the researcher and asked if they would complete the questionnaire. Participation was entirely voluntary, and no reward was offered for participating. Following consent, participants filled in the questionnaires online for ease of processing, but in the presence of a researcher to ensure that they did not make attempts to prepare for or find out answers to questions about ASC. They were reassured that they were not expected to know the answers to all questions but to answer as best they could. The instructions also informed participants that the term “ASD” would be used throughout the questionnaire (consistent with DSM-5 terminology) but that this refers to the condition sometimes described as “autism”. The entire procedure received ethical approval from the School of Psychology Ethics Committee at University of Nottingham UK and University of Nottingham Malaysia Ethics Committee.

### Measures

Beliefs about aetiology and treatment of ASC were measured with a 24 item questionnaire (Furnham and Buck 2003), scored on a 7-point Likert scale (1 = very inaccurate to 7 = very accurate), with the only amendment being the change in terminology from ‘autism’ to ‘Autism Spectrum Disorder’ for the present study. The questionnaire was originally developed to focus on a range of different types of theories about the causes of autism including biomedical and psychogenic theories, and behavioural theories about the treatment of autism.

Knowledge about ASD was measured with a list of 12 features, six describing ASD and six describing other disorders according to the DSM-5 (American Psychiatric Association 2013). Participants had to rate the accuracy of the statements with respect to ASD on a 7-point Likert-scale (1 = very inaccurate to 7 = very accurate). However, for the purpose of scoring this questionnaire, the ratings were converted to dichotomous scores of either 1 or 0. For the items that were accurate features of ASD, if they were rated as 5, 6 or 7, a score of 1 was given, while a score of 0 was given if the item was rated 1–4. For items that were not features of ASD ratings of 1, 2 or 3 were replaced with 1 and ratings of 4–7 were replaced with 0.

To measure the quantity of past contact with someone with ASC, we used the—for ASC adapted version (Gardiner and Iarocci 2014) of—the Level-of-Contact report (Holmes et al. 1999). This report describes 12 ranked levels of exposure to ASC (the highest rank being “I have an ASD”). Participants had to endorse all items that applied to their own situation. For scoring, each item was assigned a rank and the participant’s total score was calculated by summing the ranks of the items that were endorsed.

The quality of contact was measured with one additional item: “Overall my experiences with ASD individuals have been positive”, rated on a 7-point Likert-scale (1 = strongly disagree to 7 = strongly agree). Participants rated this statement only if they had contact with someone with ASC, as indicated on the Level-of-Contact report.

Acceptance was measured with an eight-item scale previously used by Griffin et al. (2012). This asked the participant about a hypothetical person with ASC on their course. For instance, “Would you help them find a building on campus?”; “Would you introduce them to your friends?”; “Would you invite them to an activity outside of class?” Participants rated their response on a Likert-scale (1 = no, 3 = maybe and 5 = yes). The responses for each item were added up to produce an overall score out of 40.

The questionnaire ended with some demographic information about the students, such as nationality, gender, study subject.

The willingness to interact with someone with ASC was measured with a covert measure similar to the one used by Gardiner and Iarocci (2014), though it was amended to account for willingness to interact as opposed to intention to volunteer with individuals who have ASC. This consisted of a message about a visit to an ASC centre where participants could interact with individuals with ASC. Participants were asked to provide their email address if they were interested in attending the centre. During debriefing it was explained to participants that their response to this question was to be used as additional data in the analysis, and they were reminded of their right to withdraw. This measure was scored dichotomous; including an email address was treated as “yes”, and not including an email address was treated as a “no” response.

### Statistical Analyses

A principal components analysis (PCA) was conducted on the autism beliefs measure in order to identify clusters of associated beliefs about ASC. PCA using orthogonal extraction (varimax) was used to provide easily interpretable factors. Individual participants’ scores on the resulting factors were used in the subsequent analyses.

Independent samples t-tests were used compare the groups on the belief factors, ASD knowledge, quantity and quality of contact and acceptance measures, while Chi square contingency tests were used to investigate the association between group and the intention to interact with autistic people (whether or not the participant left his/her email address).

A series of mediation analyses were carried out to examine whether the relationship between participant group and acceptance of ASC was mediated by, beliefs, knowledge, quality and quantity of contact, and then whether these variables and acceptance of ASC mediated group differences in intention to interact with autistic people. The analyses were conducted using the PROCESS macro in SPSS (Hayes 2012), using models number 4 and 80.

## Results

One participant from the UK was removed due to a large amount of missing data (i.e., ASD knowledge). The remaining data set had a very small amount of missing data (< 0.01%), which affected three of the measures: ASC beliefs, ASD knowledge, and acceptance. For each of these measures, missing data was replaced using expectation–maximisation method.

### ASC Beliefs

Bartlett’s test of sphericity was significant, *χ*^2^(276) = 2790.14, *p* < .001, and the Kaiser–Meyer–Olkin measure indicated adequate sampling accuracy (KMO = 0.78). Eight factors had eigenvalues of more than 1. However, on inspecting the scree plot, there was a clear point of inflection at 6 factors so the PCA was re-run extracting 5 factors in total. This set of factors explained 52.97% of the variance in the data. Table 2 shows factor loadings after rotation.

**Table 2:** The factors from the 5-factor model of beliefs about ASD, and loadings of the items on these factors

Item	Loading
Factor 1: Upbringing (% of variance = 16.12) Cronbach’s α = 0.81	
16 Cold and unloving homes are a frequent cause of ASD	0.82
9 Having a ‘bad upbringing’ causes ASD	0.81
8 Having emotionally cold parents often causes ASD	0.78
5 Traumatic experiences early in life can cause ASD	0.65
24 Treatment of ASD is easier if the sufferer really wants to get better	0.55
19 A belief in God can help a person overcome ASD	0.51
3 ASD is most often caused by illness during pregnancy	0.43
Factor 2: Biological causes (% of variance = 11.99) Cronbach’s α = 0.75	
4 Brain abnormalities are the main cause of ASD	0.75
22 The main cause of ASD is brain abnormality	0.73
11 ASD is caused essentially by genetic factors	0.68
21 ASD is passed to children through genes	0.65
20 Complications during pregnancy can cause ASD	0.60
3 ASD is most often called by illness during pregnancy	0.46
2 A chemical imbalance is the main cause of ASD	0.43
Factor 3: Interventions (% of variance = 8.97) Cronbach’s α = 0.66	
18 Drugs are an effective way of treating ASD	0.79
10 The best way to treat ASD is using appropriately prescribed drugs	0.76
17 Punishing ‘strange’ and inappropriate behaviour can reduce ASD	0.54
14 Whether a person with ASD gets better may simply depend on luck	0.50
15 Giving ‘rewards’ for ‘normal’ behaviour can reduce ASD behaviour	0.44
Factor 4: Food (% of variance = 8.43) Cronbach’s α = 0.73	
12 Changes in diet can be very effective in treating ASD	0.83
13 Eating certain types of food can worsen ASD behaviour	0.82
23 Allergies to some foods can cause ASD	0.66
Factor 5: Supportive environment (% of variance = 7.46) Cronbach’s α = 0.55	
7 Providing a warm and loving environment can help people overcome ASD	0.75
1 Individuals with ASD can be helped to improve their behaviour through one-to-one therapy	0.73
6 ASD can best be helped by encouraging sufferers to interact with others who are ‘normal’	0.53

### Group Comparisons

Independent samples t-tests showed that there were significant differences in beliefs about Upbringing and Food, ASD knowledge, quantity and quality of contact and acceptance (*p *<* .*01; see Table 3).

**Table 3:** Mean, standard deviation, and comparison for UK and Malaysian campuses on the ASD beliefs factors, knowledge of ASD, quantity and quality of contact with, and acceptance of autistic individuals

	UK (*N *= 166)	Malaysia (*N *= 195)	Significance
Beliefs			
Upbringing	− 0.37 (0.93)	0.31 (0.95)	*t*(359) = 6.79 ***
Biological causes	0.11 (0.94)	− 0.09 (1.04)	*t*(359) = 1.96
Interventions	0.04 (1.02)	− 0.03 (0.98)	*t*(359) = 0.63
Food	1.51 (1.02)	− 0.13 (0.97)	*t*(359) = 2.68 **
Supportive environment	0.05 (0.95)	− 0.04 (1.04)	*t*(359) = 0.92
Knowledge of ASD features	7.05 (2.11)	5.87 (2.21)	*t*(359) = 5.17 ***
Quantity of contact	12.35 (10.62)	6.96 (6.69)	*t*(359) = 5.66 ***
Quality of contact	5.14 (1.23)	4.54 (1.02)	*t*(240) = 4.11 ***
Acceptance	35.86 (4.40)	33.33 (5.81)	*t*(359) = 4.70 ***
Willingness to interact	N = 76 (45.8%)	N = 72 (36.9%)	*χ*^2^(1) = 2.91

**p < .01

***p < .001

To determine whether differences in the field of study (Arts & Humanities, Social sciences & Law, Science & Medicine, Engineering), between the two campuses were responsible for these differences, one-way ANOVAs were carried out comparing scores on each variable for the different fields of study, at each campus. At the UK campus, there was a significant effect of field of study on knowledge of ASD features only *F*(3151) = 6.15, *p* < .01, (Science & Medicine students scoring significantly higher than Social sciences & Law (*p* < .01) and Engineering (*p* < .05) students. At the Malaysia campus, there was an effect of field of study on the Biological causes beliefs factor, *F*(3184) = 6.50, *p* < .001. Engineering students scored higher than students studying Arts & Humanities (*p* < .05) or Science (*p* < .001). On all other variables, there was no difference across fields of study at either campus.

Students studying psychology made up a proportion of the sample at both campuses (UK *N* = 23, 13.9%; MY *N* = 35, 18.5%). As these students probably studied ASC as part of their degree course and therefore could be expected to have greater knowledge of ASD, the above analyses were repeated with students studying psychology removed from the sample. The pattern of group differences for this restricted sample was identical to that in Table 3.

The same analyses were repeated including only the British (*N* = 111) and Malaysian (*N* = 133) participants. This analysis revealed broadly similar findings with two differences. Firstly, the British participants (*M* = 0.10, *S.D.* = 0.84) scored significantly higher than the Malaysian participants (*M* = − 0.22, *S.D.* = 1.06) on the Biological causes beliefs factor, *t*(242) = 2.53, p < .05. Secondly, the British participants (*M* = 0.08, *S.D.* = 1.02) scored significantly higher than the Malaysian participants (*M* = -0.19, *S.D.* = 0.95) on the Interventions beliefs factor, *t*(242) = 2.16, *p* < .05. All other comparisons were the same as those displayed in Table 3.

Seventy-six participants (45.8%) from the UK campus and 72 participants (36.9%) from the Malaysian campus left their contact details (Willingness to interact). The group difference was not significant, *χ*^2^(1) = 2.91, *p *> .05. When including only the British and Malaysian students, 43 British (38.7%) and 54 Malaysian participants (40.6%) left their contact details. The group difference was again not significant, *χ*^2^(1) = 0.09, *p* > .05. There was no association between field of study and likelihood of providing contact details at the UK campus. However, at the Malaysia campus, there was a significant association, *χ*^2^(3) = 12.52, *p* < .01. Students studying science (52.6%) were more likely to leave their details than students studying engineering (23.3%), *χ*^2^(1) = 9.73, *p* < .01, or social science (29.8%), *χ*^2^(1) = 6.92, *p* < .01. Excluding the psychology students, 61 participants (42.7%) in the UK campus sample and 45 participants (28.3%) in the Malaysia campus sample left their contact details. This group difference was significant, *χ*^2^(1) = 6.81, *p* < .01.

To explore whether studying psychology might negate some of these cross-cultural differences, we carried out the same analyses with just the psychology students, although the resulting samples were rather small, with 23 UK based students and 36 Malaysia based students. Comparisons revealed that psychology students in Malaysia (*M *= − 0.04, *S.D.* = 1.14) scored higher than psychology students in the UK (*M* = − 0.68, *S.D.* = 0.67) on the beliefs about Upbringing factor, *t*(57) = 2.73, *p* < .01, but there were no group differences on the other four factors. UK based psychology students (*M* = 8.74, *S.D.* = 1.45) scored higher than Malaysia based psychology students (*M* = 7.06, *S.D.* = 2.56) on ASD knowledge, *t*(57) = 3.22, *p* < .01. UK psychology students (*M* = 13.13, *S.D.* = 8.97) also scored higher than psychology students in Malaysia (*M* = 7.61, *S.D.* = 8.19) on quantity of contact, *t*(57) = 2.43, *p* < .05, but there were no group differences in quality of contact or acceptance. Fifteen psychology students (65.2%) in the UK and 27 psychology students (75.0%) in Malaysia left their email address, indicative of willingness to interact. This group difference was not significant.

To summarise, overall the pattern of results was similar across students studying in different fields and regardless of whether psychology students were included or excluded from the sample. There was one notable exception; when psychology students were excluded, students at the Malaysia campus were less likely to leave their email address (willingness to interact). When considering British and Malaysian students only, there were differences in some of the beliefs factors, but aside from this, the pattern of results was the same as for the whole sample.

### Factors Predicting ASC Acceptance

**Fig. 1:**
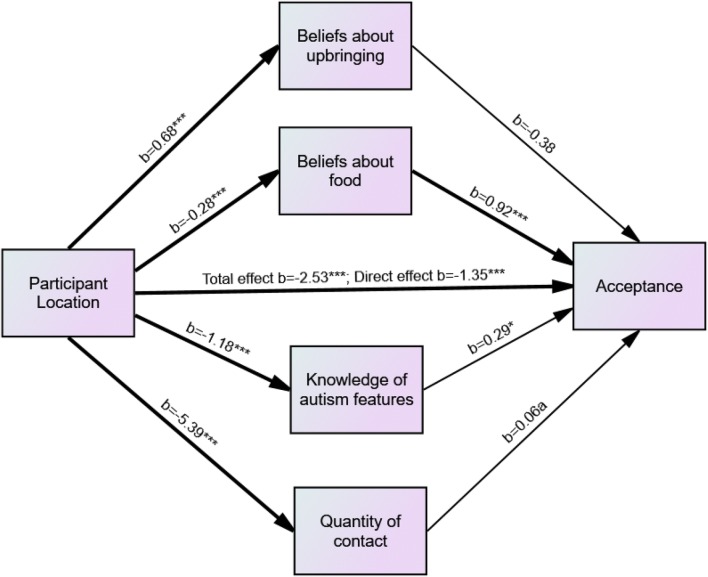
Model of the relationship between participant location and acceptance, with knowledge of autism features, quantity of contact and beliefs about ASD as mediators. *p < .05, **p < .01, ***p < .001, ^a^p = .051

Mediation analysis was conducted to determine whether the observed difference in acceptance of ASC for participants in the UK and Malaysia was mediated by the observed differences in beliefs, knowledge and quantity of contact. The independent variable in the model was participant group (UK or Malaysia), the dependent variable was acceptance of ASC, and the mediator variables were beliefs about upbringing and food factors (as these differed significantly between groups), knowledge of ASD, quantity of contact (see Fig. 1).

Although participants at the Malaysia campus had higher scores on the beliefs about upbringing factor than participants at the UK campus, *b* = 0.68, *p* < .001, this was not a significant predictor of acceptance. A 95% bias-corrected confidence interval based on 5000 bootstrap samples indicated that the indirect effect of participant group through beliefs about upbringing (− 0.25), holding all other mediators constant, was not different from zero (− 0.77 to 0.24). Participants at the UK campus scored higher on beliefs about food, *b* = − 0.28, *p* < .001, and this in turn was a significant predictor of acceptance of ASC, *b* = 0.92, *p* < .001. A 95% bias-corrected confidence interval based on 5000 bootstrap samples indicated that the indirect effect of participant group through beliefs about food (− 0.26), holding all other mediators constant, was entirely below zero (− 0.5 to − 0.06).

Participants at the UK campus had a greater knowledge of ASD features than those at the Malaysia campus, *b* = − 1.18, *p* < .001, and knowledge of ASD features in turn predicted acceptance of ASC, *b* = 0.29, *p* < .05. A 95% bias-corrected confidence interval based on 5000 bootstrap samples indicated that the indirect effect of participant group through knowledge of ASD features (− 0.34), holding all other mediators constant, was entirely below zero (− 0.71 to − 0.02).

Participants at the UK campus reported having had more contact with ASC individuals, *b* = − 5.39, *p* < .001 and contact was a marginally significant predictor of acceptance of ASC, *b* = 0.06, *p* = .051. A 95% bias-corrected confidence interval based on 5000 bootstrap samples indicated that the indirect effect of participant group through quantity of contact (− 0.33), holding all other mediators constant, was entirely below zero (− 0.65 to − 0.01).

UK campus participants still reported greater acceptance of ASC even when the effects of all mediators were taken into account, *b* = − 1.35, *p* < .001.

**Fig. 2:**
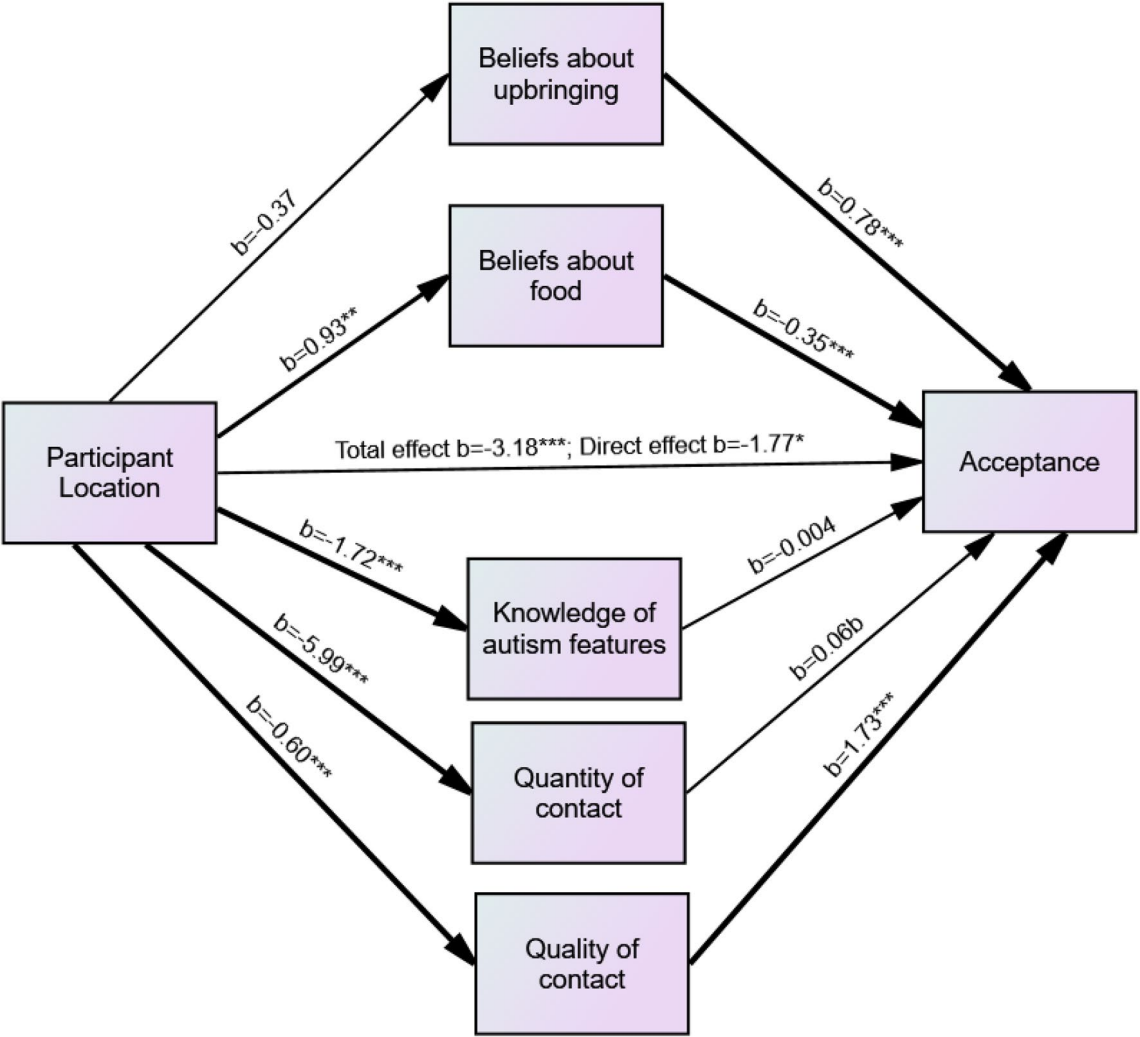
Model of the relationship between participant location and acceptance, with knowledge of autism features, quantity and quality of contact, and beliefs about ASD as mediators. *p < .05, **p < .01, ***p < .001, ^b^p = .056

A second mediation analysis was carried out including only those participants who reported having had direct contact with autistic individuals (UK *N* = 118, 71.1%; MY *N* = 124, 63.4%), which included the additional mediator of quality of contact. The independent variable in the model was participant location (UK or Malaysia), the dependent variable was acceptance of ASC, and the mediator variables were beliefs about upbringing and beliefs about food factors, knowledge of ASD features, quantity of contact, and quality of contact (see Fig. 2).

Although participants at the Malaysia campus had higher scores on the beliefs about upbringing factor than participants at the UK campus, *b* = 0.78, p < .001, this was not a significant predictor of acceptance. A 95% bias-corrected confidence interval based on 5000 bootstrap samples indicated that the indirect effect of participant group through beliefs about upbringing (− 0.29), holding all other mediators constant, was not different from zero (− 0.23 to 0.87).Participants at the UK campus scored higher on beliefs about food, *b* = − 0.35, *p* < .001, and this in turn was a significant predictor of acceptance of ASC, *b* = 0.93, *p* < .01. A 95% bias-corrected confidence interval based on 5000 bootstrap samples indicated that the indirect effect of participant group through beliefs about food (− 0.32), holding all other mediators constant, was entirely below zero (− 0.69 to − 0.07).

Participants at the UK campus had a greater knowledge of ASD features than those at the Malaysia campus, *b* = − 1.72, *p* < .001, but knowledge of ASD features did not significantly predict acceptance of ASC. A 95% bias-corrected confidence interval based on 5000 bootstrap samples indicated that the indirect effect of participant group through knowledge of ASD features (0.01), holding all other mediators constant, was not different from zero (− 0.54 to − 0.57).

Participants at the UK campus reported having had more contact with autistic individuals*, b* = − 5.99, *p* < .001 and contact was a marginally significant predictor of acceptance of ASC, *b* = 0.06, *p* = .056. A 95% bias-corrected confidence interval based on 5000 bootstrap samples indicated that the indirect effect of participant group through quantity of contact (− 0.73), holding all other mediators constant, was not different from zero (− 0.78 to 0.03).

Participants at the UK campus also reported having had better quality contact with autistic individuals, *b* = − 0.60, *p* < .001 and quality of contact was a significant predictor of acceptance of ASC, b = 1.73, p < .001. A 95% bias-corrected confidence interval based on 5000 bootstrap samples indicated that the indirect effect of participant group through quantity of contact (− 1.03), holding all other mediators constant, was entirely below zero (− 1.78 to − 0.46).

UK campus participants still reported greater acceptance of ASC even when the effects of all mediators were taken into account, *b* = − 1.77, *p* < .05.

Again, this analysis was repeated including only the British and Malaysian students in the sample. Beliefs about biological causes of ASC and beliefs about interventions were also included as potential mediators as they differed for British and Malaysian participants. The final model revealed that quantity of contact (b = − 0.63, CI − 1.15, − 0.17), beliefs about food (b = − 0.41, CI − 0.85, − 0.08) and quality of contact (b = − 1.02, CI − 1.91, − 0.38), were significant mediators of the relationship between participant group and acceptance. British participants still reported greater acceptance of ASC even when the effects of all mediators were taken into account, *b* = − 1.80, *p* < .05.

### Factors Predicting Willingness to Interact

**Fig. 3:**
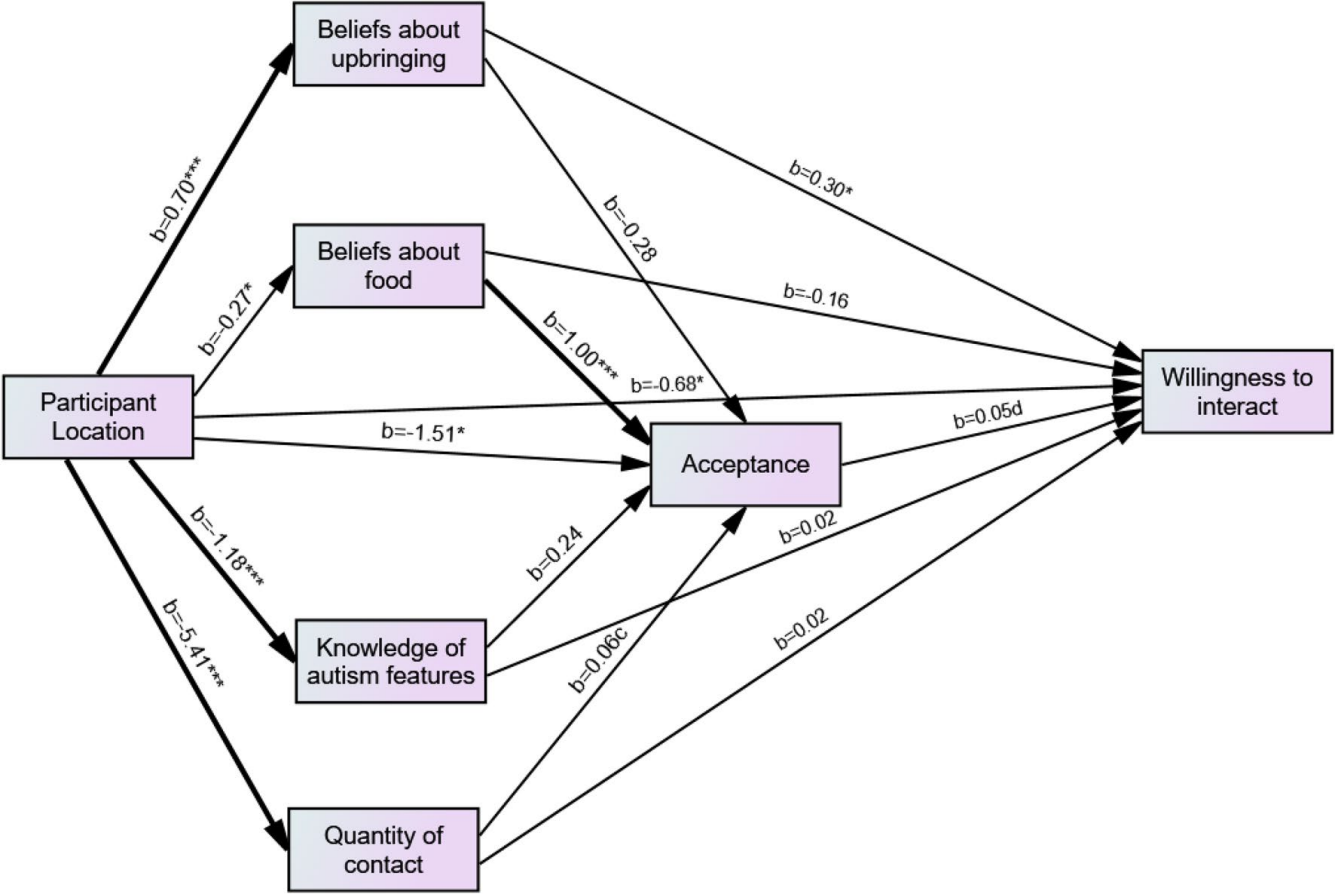
Model for the relationship between participant location and willingness to interact with those with ASD, with knowledge, of autism features, quantity of contact, beliefs about ASD, and acceptance as mediators (psychology students were excluded from this analysis). *p < .05, **p < .01, ***p < .001, ^c^p = .051, _d_p = .054

The participant groups only differed in their willingness to interact with people with ASC (as indicated by leaving their email address in the study) when psychology students were removed from the sample. Therefore a mediation analysis was conducted with psychology students excluded to determine whether the observed difference in willingness to interact for participants in the UK and Malaysia was mediated by beliefs about upbringing, beliefs about food, knowledge of autism features, quantity of contact, and, in turn, acceptance. A mediation model with parallel and sequential elements was tested (see Fig. 3). The independent variable in the model was participant group (UK or Malaysia) and the dependent variable was whether the participant entered their email address.

As illustrated in Fig. 3, whether the participants was at the UK or Malaysia campus predicted willingness to interact, *b* = − 0.68, *p* < .05. Of the other variables in the model, only beliefs about upbringing directly predicted willingness to interact, *b* = 0.30, *p* < .05 with acceptance being a marginal predictor, *b* = 0.05, *p* = .054.

Analyses examining possible mediating effects revealed that, although participants at the Malaysia campus had higher scores on the beliefs about upbringing factor than participants at the UK campus, *b* = 0.70, *p* < .001, this was not a significant predictor of acceptance. A 95% bias-corrected confidence interval based on 5000 bootstrap samples indicated that the indirect effect of participant group through beliefs about upbringing and acceptance (− 0.01), holding all other mediators constant, was not different from zero (− 0.05 to 0.02). Participants at the Malaysia campus scored higher on the beliefs about upbringing factor and beliefs about upbringing predicted whether participants left their email addresses (willingness to interact). However, the indirect effect of participant location (UK or Malaysia) on willingness to interact via beliefs about upbringing (0.21) holding all other mediators constant was not different from zero (0 to 0.48). Participants from the UK campus were more likely to leave their email address even when the effects of all mediators were taken into account, *b* = 0.68, *p* < .05.

Participants at the UK campus scored higher on beliefs about food, *b* = − 0.27, *p* < .05, and this in turn was a significant predictor of acceptance of ASC, *b* = 1.00, *p* < .001. But a 95% bias-corrected confidence interval based on 5000 bootstrap samples indicated that the indirect effect of participant group through knowledge of autism features and acceptance (− 0.01), holding all other mediators constant, was not different from zero (− 0.04 to 0).

Participants at the UK campus had a greater knowledge of ASD features than those at the Malaysia campus, *b* = − 1.18, *p* < .001, but knowledge of ASD features did not significantly predict acceptance of ASC. Moreover, a 95% bias-corrected confidence interval based on 5000 bootstrap samples indicated that the indirect effect of participant group through knowledge of autism features and acceptance (− 0.01), holding all other mediators constant, was not different from zero (− 0.05 to 0). Participants at the UK campus reported having had more contact with autistic individuals, *b* = − 5.41, *p* < .001 and contact was a marginally significant predictor of acceptance of ASC, *b* = 0.06, *p* = .051. However, a 95% bias-corrected confidence interval based on 5000 bootstrap samples indicated that the indirect effect of participant group through quantity of contact and acceptance (− 0.02), holding all other mediators constant, was not different from zero (− 0.06 to 0).

## Discussion

Social interaction is of utmost importance for any individual. Individuals with ASC have difficulties with social interaction (American Psychiatric Association 2013). Individuals without ASC having trouble to interpret mental states of individuals with ASC (Sheppard et al. 2016) might compound these difficulties. Culture might influence beliefs and knowledge about ASD, and this in turn might influence acceptance of and willingness to interact with someone with ASC. We aimed to study whether two countries with different cultures; the UK and Malaysia, differed with respect to knowledge and beliefs about ASC, contact with those with ASC, and how these influence acceptance and willingness to interact with someone with ASC.

Beliefs about ASC were assessed using a questionnaire developed in the UK (Furnham and Buck 2003). In the current study, five factors emerged; Upbringing (bad upbringing might cause ASC), Biological causes (ASC has a biological cause), Interventions (ASC can be alleviated with interventions), Food (ASC is related to diet), and Supportive environment (a positive environment can alleviate ASC traits). Of these factors, the Upbringing factor was populated by similar items to those in the first factor extracted in Furnham and Buck’s analysis, which they had named Psychogenic and external. Likewise, the fourth factor extracted in both analyses was a Beliefs about food (diet) factor and included the same items in each study. However, the other three factors that were extracted in each study were somewhat different. For instance, the biological causes factor (Factor 2 in the current study) was populated by items that were distributed across the three remaining factors in Furnham and Buck’s study. The Interventions factor in the current study consisted of some items which appeared in the third factor in Furnham and Buck’s study (named Genes and drugs) but also items that formed part of their first factor (Psychogenic and external). Finally, the Supportive environment factor from the current study consisted of two items from factor 2 (named Pregnancy and environmental treatment) in Furnham and Buck’s study. These slight differences in factor structure might be due to the inclusion of participants located in Malaysia in the current study, but there are other differences between the studies that could be at play. Firstly, the current research used a student sample whereas Furnham and Buck (2003) sampled the wider population. Secondly, Furnham and Buck’s study took place some 15 years earlier so the differences could reflect genuine shifts in understanding of ASC over that timeframe. In fact, knowledge about and attitudes towards ASC appear to shift even in a relatively brief period of 5 years (White et al. 2019). Finally, Furnham and Buck provided their participants with a brief description of autism before administering the questionnaire, whereas we did not, which could have impacted their beliefs.

Three of the five identified factors did not differ between students based in the UK and Malaysia. Students on both campuses showed similar levels of belief that ASC has a biological basis, had similar views on the utility of interventions in reducing autistic features, and believed to a similar extent that providing social support can help people with ASC. Differences between the two campuses were observed on the other two factors. UK based students scored higher on the food factor, which was underpinned by statements that suggested that food intolerances could cause ASC, influence its presentation, and that dietary interventions could reduce autistic features. In contrast, Malaysian based students scored higher on the upbringing factor, which primarily consisted of items suggesting that negative experiences early in life or poor parenting can be a cause of ASC. These differences appear to be consistent with respondents in the UK having an understanding of ASC that is more in line with current research. In recent years, there has been an increasing research focus on the role of diet in managing behaviour in ASC, with a growing number of studies addressing gluten-free or casein-free diets in ASC (e.g. Millward et al. 2008; Pennesi and Klein 2012; Whiteley et al. 1999). By comparison, the suggestion that ASC results from a particular kind of upbringing has been discredited for many years within the research community (e.g. Ramachandran and Oberman 2006).

When analysis was restricted to only students of British or Malaysian nationality, the differences in scores on the food and supportive environment factors were retained. However, British participants were also found to score higher on the beliefs in biological causes factor as well as on the beliefs in interventions factor. In both of these cases, this difference appears to be due to the mean scores of the Malaysian students on these factors being particularly low compared with students at the Malaysian campus in general. It is not immediately clear why this is the case. Although one might predict a greater understanding of the biological basis of the interventions used for ASC in a western sample, it is surprising that this effect appeared only when British and Malaysian students were compared. Knowledge of ASD features was assessed in this study by asking participants to make a judgment about which of a series of diagnostic characteristics taken from DSM-5 were features of ASD and which were not. Students in the UK scored higher on this measure, indicating, as predicted, a greater knowledge of ASD according to current diagnostic criteria. These differences were maintained when the narrower comparison was made between British and Malaysian students only and when psychology students (who may have studied ASD as part of their course) were excluded from the analysis. Furthermore, UK based students reported having had more contact with autistic individuals, as well as that contact being more positive than students based in Malaysia. This pattern also emerged when only British and Malaysian nationality students were considered and regardless of whether psychology students were included or excluded from the samples. UK-based students reported greater acceptance of ASC than Malaysia-based students and, again, this difference was maintained when considering just the British and Malaysian nationality students and when psychology students were excluded.

The difference in knowledge and acceptance might be a result of generally poorer awareness of ASC in Malaysia resulting in reduced knowledge of and exposure to autistic individuals in this context. This might be related to stigma and mental healthcare in Malaysia. Up until recent years, there has been considerable stigma about mental health in Malaysia (Toran et al. 2011). Moreover, mental health care facilities are often either only accessible for the more fortunate, or have long wait lists (Neik et al. 2014). The combination of stigma and low access to mental health care facilities might lead to late diagnoses, and not being open about a diagnosis such as ASD. As a result, people might be less aware of the existence and features of ASD. Luckily, there is a large increase in autism centres in the recent years. Moreover, the government has successfully urged health care insurance companies to cover mental health (Aqilah 2019). The more negative experiences and lower acceptance of ASC reported by Malaysia based students might be related to stigma associated with ASC in this population.

Willingness to interact with someone with ASC was assessed via a covert behavioural measure whereby participants were offered the opportunity to visit a local autism centre in order to meet individuals with ASC. Participants were asked to enter their email address if they were interested in visiting the centre, indicating a desire to interact with those with ASC. There was no cross-cultural difference in willingness to interact when the sample was considered as a whole, or when only British and Malaysian students were compared. However, when psychology students were excluded (who had a high tendency to leave their email address across both campuses), the remaining participants at the Malaysia campus were less likely to leave their contact details than those in the UK. The sample with psychology students excluded can probably be considered more representative of the views of the student bodies as a whole, and thus, the behavioural measure supports the notion that, in line with their expressed attitudes, those in Malaysia are less positive towards autism at a behavioural level.

Additionally, we compared only the psychology students in the UK and Malaysia on all these variables, and found the same differences in beliefs about upbringing, knowledge of ASD features, and quantity of contact. However, unlike in the full sample, no differences were found in quality of contact or acceptance. These findings should be interpreted with caution given the small sample size, which might contribute to the lack of significant effects. However, tentatively, these results might imply that although studying psychology does not eradicate cross-cultural differences in knowledge and experience of ASC, it does result in more positive attitudes towards people with ASC, in the sense of quality of contact and acceptance. Alternatively, it could be that those who have had good quality of contact and acceptance of people with ASC (or other psychological diagnoses), might be more inclined to study psychology. In the future, it would be informative to replicate this study with a larger sample of psychology students, and in particular to question which factors (if not ASD knowledge and direct contact) mediate more positive attitudes in Malaysia based psychology students, as in psychology students, positive attitudes seem even more important than in other students.

For the whole sample, a series of mediation analyses were conducted to determine whether the observed differences in acceptance and willingness to interact with those with ASC for participants in the UK and Malaysia could be explained by the differences in beliefs about autism, knowledge of autism features, quantity and quality of contact. These showed that the differences in acceptance of ASC at the two campuses could be partly explained by differences in knowledge of ASD features and the amount of contact with individuals with ASC in the two locations. Beliefs about food were also a significant mediator of the relationship between participant location and acceptance. While at first this seems surprising, if it is the case that endorsing the statements in relation to beliefs about food is indicative of a greater awareness of current research about autism (as discussed previously), this might in turn explain the relationship. When analyses focused on those participants who had had direct contact with those with ASC, the amount of contact no longer mediated the relationship between location and acceptance, but the quality of contact did, while beliefs about food remained a significant mediator. While significant mediation was found to occur, the relationship between participant location and acceptance remained even when the effects of all mediators were taken into account. This indicates that there are potentially other reasons why acceptance differed for UK and Malaysia based students that go beyond the knowledge, contact and beliefs variables that were measured. One possibility might be that it is less socially desirable to express negative attitudes to ASC in the UK than Malaysia—or conversely that it may be socially undesirable to express positive attitudes to ASC in Malaysia. This could result in a cross-cultural difference that is unrelated to the constructs of knowledge, beliefs and contact that were measured.

Possible mediators of the relationship between participant location and willingness to interact were investigated with psychology students excluded from the sample, as no group differences were observed when psychology students were included. However, none of the variables considered in the analysis were found to be significant mediators. This contrasts with the findings of Gardiner and Iarocci (2014) who found a strong relationship between the same acceptance measure and a similar behavioural measure in their sample in Canada, although they did not do a mediation analysis. The lack of mediation here indicates that some other aspect of difference between participants at the UK and Malaysia campuses is responsible for the difference in willingness to interact. It is unlikely that socially desirable responding could account for the effect as the invitation to visit the autism centre was presented as unrelated to the rest of the research project and participants were unaware that their responses to this question would be analysed. The cross-cultural difference could potentially reflect differences in logistical constraints for the participants at the two campuses making participants in Malaysia less likely to leave their email addresses for reasons totally unrelated to their feelings about autism. However, if this was the case we might expect that even among psychology students, fewer in Malaysia would have left their contact details, which did not happen.

Specific Asian/Malaysian cultural values might partly explain a number of our findings. As we have not studied this directly, these are merely surmises. Firstly, the collective (Hoftede et al. 2010), family centred Malaysian culture might explain the perceived link between ASC and upbringing, and lower level of acceptance. A collectivistic culture leaves less room to deviate from the norm. While in Western cultures standing out from the crowd is encouraged, in Asia, children are expected to be quiet, disciplined, not make waves or inconvenience others (Kim et al. 1999). Deviating from the norm, such as behavioural or psychological problems, is less accepted, is expected to be handled within the family (Toran et al. 2011), and might be blamed on the family. Moreover, people are expected to resolve psychological problems on their own (Kim et al. 1999). Going to an autism centre might be considered intervening in someone’s personal and family life, which could explain why Malaysian students are less eager to visit an autism centre. Psychology students, on the other hand, learn about the effectiveness of professional interventions for psychological conditions, and might, therefore, be *more* inclined to visit an autism centre. Secondly, the strong study focus in Asia (Kim et al. 1999) might influence willingness to interact. Although for psychology students visiting an autism centre might add value to their studies, non-psychology students might prefer to focus on their study or visit study relevant places. Thirdly, that food is not strongly linked to ASC in Malaysia might result from the prominent role food plays. Food is considered a bonding factor between the different cultures and ethnicities (Perry 2017), plays a large social role, and it is an important tourist attraction (Zainal et al. 2010). Although in food choice (physical) Health, Natural Content, and Weight Control are considered, psychological factors such as Mood are not strongly considered in Malaysia (Prescott et al. 2002). Moreover, the wisdom from elderly is highly valued in Asia (Kim et al. 1999), and elderly seem less open to try new foods (Prescott et al. 2002), and possibly diets. In short, food plays an important (social) role in Malaysia, but is not linked to psychological problems. Finally, the lower level of contact with people with ASC in Malaysia might result from the lower number of diagnoses overall, and a lack of inclusive education. Despite a positive attitude from teachers towards inclusive education (Ali et al. 2006; Bailey et al. 2015), the resources to implement this on a broad level are limited (Nasir and Efendi 2017). Students might hence not encounter peers with special needs in an educational setting or might not be aware of the diagnosis a peer might have. As a result, people with an ASC might have less access to mainstream education, be less accepted, and interact less with people outside of the family. Importantly, we did not study these effects explicitly, and future endeavors might give more insight in the relation between specific cultural values and knowledge about, acceptance of, and willingness to interact with individuals with ASC.

Our findings give interesting insights in differences between students’ beliefs and attitudes towards ASC in European and Asian cultures and countries. However, the current sample is not representative for the general population; (1) Only students were included, hence highly educated and specifically in Malaysia from a high socio-economic background, (2) Both campuses are in urban areas (although the students might not originate from these areas). People who are lower educated might stigmatise more as compared to higher educated people (Milačić-Vidojević et al. 2014). Moreover, knowledge and beliefs might be different when people are from rural areas, and from a lower socioeconomic background (Toran et al. 2011).

Our findings show that, in two different cultures, beliefs about ASC differed in some, but not all respects; UK based students believed more often that ASC was food related, in line with recent research, and Malaysia based students more often believed that upbringing and early life might be related to ASC. Additionally, students in the UK had more knowledge, contact, positive experiences, and acceptance of ASC. When psychology students were excluded from analyses, UK students were more willing to interact with autistic individuals. Differences in knowledge, and contact explained (partly) the difference in acceptance, as well as the belief that ASC is food related. However, acceptance did not seem to mediate the relation between country, beliefs, knowledge, and experience and willingness to interact. This indicates that knowledge about and contact with people with ASC might improve acceptance in different cultures. However, how acceptance would lead to more willingness to interact has to be explored in future research.
